# Editorial: Bio-Inspired Nanomaterials in Surface Engineering and Bioapplications

**DOI:** 10.3389/fchem.2022.872069

**Published:** 2022-03-14

**Authors:** Yun Jun Yang, Zhong Feng Gao

**Affiliations:** ^1^ Advanced Research Institute for Multidisciplinary Science, Qilu University of Technology (Shandong Academy of Sciences), Jinan, China; ^2^ Advanced Materials Institute, Qilu University of Technology (Shandong Academy of Sciences), Jinan, China; ^3^ Collaborative Innovation Center of Tumor Marker Detection Technology, Equipment and Diagnosis-Therapy Integration in Universities of Shandong, Shandong Provincial Key Laboratory of Detection Technology for Tumor Markers, College of Chemistry and Chemical Engineering, Linyi University, Linyi, China

**Keywords:** biomaterial, functional nanomaterial, biomedical application, surface chemistry, nanoscience

Bio-inspired nanomaterials, as a class of easy-to-use biomaterials, have emerged as versatile tools for biosensing, bioimaging, biocatalysis, antibacterial treatment, and biotherapy, and have demonstrated their usefulness in addressing a wide range of biomedical challenges (Huang et al., 2015; Madamsetty et al., 2019; Kumar et al., 2020; Lai et al., 2021). By using diverse chemical modification or biological interaction on the surface, the functional nanomaterial can be constructed to recognize the specific site in complex environments both *in vitro* and *in vivo*. While many biological systems require a highly specific lock-and-key approach for molecular interaction, surface engineering provides an alternative method to create specificity of biofunctional nanomaterials (Wu et al., 2016; Kankala et al., 2020). Attaching biological molecules, such as nucleic acids, proteins, and antibodies, at the surface of nanomaterials is a crucial condition to ensure the biosafety of nanomaterials. The primary feature of an advanced nanomaterial is that it provides low toxicity, high biocompatibility, and unique stimulus responses such as light, electricity, and magnetism, which expand its bioapplications (Reddy et al., 2012; Ge et al., 2021; Li et al., 2021).

To present state-of-the-art research in this field, we launched a Research Topic in Frontiers in Chemistry entitled “Bio-Inspired Nanomaterials in Surface Engineering and Bioapplications.” This Research Topic included 11 articles, including 6 original research articles, 3 reviews, and 2 mini reviews, which covered the field of surface treatment, biosensors, tissue engineering, cancer therapy, and other bioapplications.

Several works focus on the surface engineering of nanomaterials because new surface chemistry represents a revolutionary direction in functional biomaterials and has become a topic of interest. Liu et al. reviewed the significant progress in the surface functionalities of metallic implants regarding their physical structure, chemical composition, and biological reaction by surface treatment and bioactive coating. They have presented a perspective on the current challenges and future directions for development of surface treatment on 3D-printed implants. Zhang et al. summarized the existing electroless plating methods for carbon nanotubes, and their applications including electrical, mechanical, thermal, tribological, corrosion resistance, and magnetic properties were discussed in detail. This review is critical for the future research and improvement of electroless metal/alloy nano-coating of carbon nanotubes.

Regarding the application examples, the integration of biomolecules or surface treatment can endow biosensors with high sensitivity and selectivity. Lin et al. developed a sensitive electrochemical biosensor using carbon dots-Fe_3_O_4_ nanomaterial (CDs-Fe_3_O_4_) for *E. coli* O157:H7 detection. The functional electrochemical biosensor showed a wide detection range from 10 to 10^8^ CFU/ml and a low detection limit (6.88 CFU/ml). This method has been successfully used to determine *E. coli* O157:H7 in milk and water samples. Meanwhile, a mini-review summarized the design principle and biosensing application of a pH-responsive DNA motif including triplex DNA, i-motif, and A^+^-C mismatch base pair-based DNA structures (Zheng et al.). They pointed out that the modification of those DNA motifs into single-cell and modulate intercellular functions will be challenging for future studies.

In the direction of tissue engineering, Han et al. introduced a nanofiber mat with dual bioactive components and a biomimetic matrix structure to improve osteogenesis capability. By combining homogeneous blending and electrospinning technology, the nanofiber mat showed sufficient mechanical properties and a porous structure suitable for cell growth and migration, which have great potential in the application of bone repair materials. By mimicking the composition of the extracellular matrix of native tissues, Xing et al. reported an injectable hydrogel tissue adhesive with excellent biocompatibility. The measured adhesion ability was higher than that of the commercial Porcine Fibrin Sealant Kit, indicating the novel injectable hydrogel might be a promising candidate for a soft tissue adhesive.

Target-triggered nanomaterials have been considered as potential delivery systems, which could achieve the targeted delivery of antitumor drugs, reduced cytotoxicity, and enhanced therapeutic efficacy. Zhou et al. used the redox-responsive star-shaped polymeric prodrug (PSSP) and the dimeric prodrug of paclitaxel (diP) to prepare a co-delivery system (diP@PSSP) for intracellular drug release in tumor cells. The redox-responsive diP@PSSP micelles possessed high drug-loading content of paclitaxel as high as 46.9% and excellent stability. The polymeric prodrug of diP@PSSP micelles also demonstrated good biocompatibility in red blood cells and had a therapeutic effect in HeLa cells. The original research article from Fang et al. reported polypyrrole (PPy)-modified Fe_3_O_4_ nanoparticles (PPy@ Fe_3_O_4_ NPs) on inhibiting growth and metastasis of non-small cell lung cancer by a combination of photothermal therapy (PTT) and chemodynamic therapy (CDT). PPy was used as a photothermal agent to construct nanocomposites because of its high photothermal conversion efficiency and exceptional photostability. The *in vitro* and *in vivo* studies displayed that PPy@Fe_3_O_4_ NPs were excellent near-infrared (NIR) sensitive magnetic resonance imaging (MRI)-guided synergistic chemodynamic/photothermal cancer therapy agents, which could decrease the levels of MMP2, MMP9, and MMP13. It provides a new therapeutic strategy for non-small cell lung cancer. As another example, Xia et al. reported a glucose oxidase (GOx)-loaded hydrogel with a pH-sensitive NIR-II photothermal effect for combinational cancer therapy at mild temperature. The hydrogels were engineered *via* coordination of alginate solution containing pH-sensitive charge-transfer nanoparticles (CTNs) as the second near-infrared (NIR-II) photothermal agents and GOx. Through consuming glucose, the hydrogel mediated starvation therapy, which not only led to exhaustion of tumor cells, but also resulted in aggravated acidity in the tumor microenvironment and downregulated expression of HSP90. By integration of mild NIR-II PTT and starvation therapy, the proposed hydrogel was able to suppress the growth of subcutaneously implanted tumors and completely prevent lung metastasis in a breast cancer murine model. Yu et al. briefly introduced the rational design, construction, and working mechanisms of NIR photoactivatable agents and summarized the recent progress of NIR photoactivatable immunomodulatory nanoparticles for combinational cancer immunotherapy. They described that NIR photoactivatable immunomodulatory nanoparticles might have great potential for clinical treatment of major diseases such as cancer, infectious diseases, and autoimmunity. Zhang et al. discussed recent developments of biomimetic nanomaterials in ferroptosis-related cancer nanomedicine. Many ferroptosis-related nano-inducers have unexpected disadvantages including low circulation time, immune exposure, and ineffective tumor targeting. Biomimetic nanomaterials may be able to provide new solutions to these limitations due to their unique physicochemical properties.

Bio-inspired nanomaterials have a wide range of applications. The contributions in this Research Topic provide various kinds of nanomaterials with unique surface treatment and bioapplications including biosensor, drug delivery, cancer therapy, and tissue engineering functions. The design, fabrication, and other applications of functional nanomaterials are still the major focus in this research field. We believe this Research Topic will provide abundant technology to understand the advanced strategies of bio-inspired nanomaterials in broad applications, inspiring novel ideas for future research fields.
